# Concentration-Dependent Effects of a Dietary Ketone Ester on Components of Energy Balance in Mice

**DOI:** 10.3389/fnut.2019.00056

**Published:** 2019-05-01

**Authors:** Sarah E. Deemer, Rachel A. H. Davis, Barbara A. Gower, Andrew P. Koutnik, Angela M. Poff, Stephanie L. Dickinson, David B. Allison, Dominic P. D'Agostino, Eric P. Plaisance

**Affiliations:** ^1^Nutrition Obesity Research Center, University of Alabama, Birmingham, AL, United States; ^2^Department of Nutrition Sciences, University of Alabama, Birmingham, AL, United States; ^3^Department of Molecular Pharmacology and Physiology, University of South Florida, Tampa, FL, United States; ^4^School of Public Health, Indiana University Bloomington, Bloomington, IN, United States; ^5^Department of Human Studies, University of Alabama, Birmingham, AL, United States

**Keywords:** ketone esters, body composition, energy expenditure, energy balance, ketogenic diet

## Abstract

**Objectives:** Exogenous ketones may provide therapeutic benefit in treatment of obesity. Administration of the ketone ester (KE) R,S-1,3-butanediol acetoacetate diester (BD-AcAc_2_) decreases body weight in mice, but effects on energy balance have not been extensively characterized. The purpose of this investigation was to explore concentration-dependent effects of BD-AcAc_2_ on energy intake and expenditure in mice.

**Methods:** Forty-two male C57BL/6J mice were randomly assigned to one of seven isocaloric diets (*n* = 6 per group): (1) Control (CON, 0% KE by kcals); (2) KE5 (5% KE); (3) KE10 (10% KE); (4) KE15 (15% KE); (5) KE20 (20% KE); (6) KE25 (25% KE); and (7) KE30 (30% KE) for 3 weeks. Energy intake and body weight were measured daily. Fat mass (FM), lean body mass (LBM), and energy expenditure (EE) were measured at completion of the study. Differences among groups were compared to CON using ANOVA and ANCOVA.

**Results:** Mean energy intake was similar between CON and each concentration of KE, except KE30 which was 12% lower than CON (*P* < 0.01). KE25 and KE30 had lower body weight and FM compared to CON, while only KE30 had lower LBM (*P* < 0.03). Adjusted resting and total EE were lower in KE30 compared to CON (*P* < 0.03), but similar for all other groups.

**Conclusions:** A diet comprised of 30% energy from BD-AcAc_2_ results in lower energy intake, coincident with lower body weight and whole animal adiposity; while KE20 and KE25 have significantly lower body weight and adiposity effects independent of changes in energy intake or expenditure.

## Introduction

Ketogenic diets (KD) decrease appetite and attenuate reductions in energy expenditure (EE) that accompany weight loss (1–3). However, ketogenesis requires near complete elimination of dietary carbohydrate resulting in poor compliance. Consumption of exogenous ketones may induce the benefits of KD while permitting more liberal consumption of carbohydrates, but research is lacking. Dietary administration of the ketone diester R,S-1,3-butanediol acetoacetate (BD-AcAc_2_) increases circulating concentrations of the ketones β-hydroxybutyrate (βHB) and acetoacetate (AcAc) in rodents (4–8), while decreasing food intake (4) and body weight (4–7). There is a paucity of information regarding the effect of BD-AcAc_2_ on energy intake and EE. The purpose of this study was to examine concentration-dependent effects of BD-AcAc_2_ on body weight and adiposity, energy intake, and EE in lean male C57BL/6J mice.

## Materials and Methods

Male C57BL/6J mice (*n* = 42) were purchased from Jackson Laboratories (Bar Harbor, ME) at 10 weeks of age and allowed to acclimate for 7 days following arrival to the UAB animal facilities. Mice were single-housed in filter-top shoebox cages on a standard 12:12 light-dark cycle in a temperature-controlled room at 22–23°C. Following acclimation, mice were randomly assigned to one of seven diets for 21 days (*n* = 6 per group, Supplement Table 1): (1) CON (0% KE), (2) KE5 (5% energy as KE), (3) KE10 (10% KE), (4) KE15 (15% KE), (5) KE20 (20% KE), (6) KE25 (25% KE), and (7) KE30 (30% KE). CON consisted of 63% carbohydrate, 17% fat, and 20% protein (Dyets Inc., #104419). Reductions in carbohydrate energy accompanied the addition of KE to produce isocaloric diets (e.g., KE5 = 5% carbohydrate energy removed and replaced with 5% KE by kcals). Mice consumed food and water *ad libitum*, and energy intake and body weight (BW) were recorded daily. The study was approved by the UAB Institutional Animal Care and Use Committee (IACUC) and all conditions conformed to the care procedures employed by the UAB Animal Resources Program.

Fat mass (FM), lean body mass (LBM), and resting (REE), and total EE (TEE) were measured in the UAB Small Animal Phenotyping Core at completion of the study. Following a 48h acclimation, EE was measured over 24h using a TSE PhenoMaster indirect calorimetry system (TSE Instruments) and body composition was measured by quantitative magnetic resonance (QMR, EchoMRI™ 3-in-1, Echo Medical Instruments). Following a 10h fast, mice were euthanized by decapitation.

Because of the small group sample size, individual KE group responses were compared only to CON using SAS (Version 9.4, Cary, NC). Differences were analyzed using PROC GLM with Dunnett's *post-hoc* test. Energy expenditure outcomes were controlled for FM and LBM. Data are expressed as mean ± standard error (SE). Significance was set *a priori* at *P* < 0.05.

## Results

### Energy Intake, Body Weight, and Body Composition

**Figure 1:**
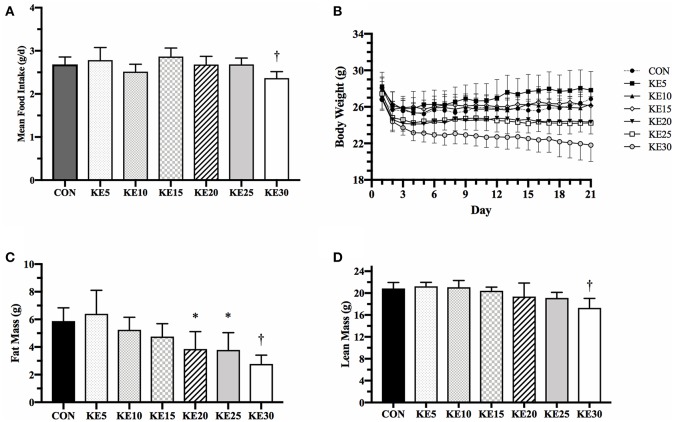
Mean food intake and body composition. **(A)** *Ad libitum* mean food intake was significantly reduced in KE30 compared to CON, but all other groups consumed a similar amount of food per day. **(B)** Body weight over the 21 days was reduced in a concentration-dependent fashion, with higher concentration of KE associated with increased reduction in body weight. **(C)** Fat mass was reduced compared to CON in KE20, KE25, and KE30, and **(D)** lean body mass was reduced only in KE30. Data are expressed as mean ± SE. ^*^Significantly different from CON (*P* < 0.05), ^†^significantly different from CON (*P* < 0.01).

Mean energy intake was the same between CON and each concentration of KE, except KE30 which was 12% lower than CON (9.0 ± 0.3 vs. 10.2 ± 0.3 kcals/d, *P* = 0.036, Figure 1A). Final BW (g), measured by QMR, was the same for KE5–KE20 compared to CON, but KE25 (23.8 ± 0.9, *P* = 0.023) and KE30 (21.0 ± 0.9, *P* < 0.001) were significantly lower than CON (27.5 ± 0.9, Figure 1B). KE20 and KE25 had lower FM (Figure 1C), but similar LBM (Figure 1D) compared to CON, while both FM and LBM was lower in KE30 compared to CON. Changes in percent body fat mirrored those of changes in fat mass, suggesting that the lower fat mass was not due to body size alone (data not shown).

### Energy Expenditure

Absolute REE and TEE were lower in both KE25 and KE30 mice compared to CON, yet, when adjusted for FM and LBM, REE, and TEE was lower only in KE30. Adjusted TEE was also lower in KE10 compared to CON (Table 1). Adjusted EE during light and dark cycle (Table 1) was not significantly different between CON and KE groups. Respiratory exchange ratio (RER) was lower in KE30 (0.79 ± 0.01) compared to CON (0.83 ± 0.01, *P* = 0.03), but similar for the other groups. There was no difference in 24*h* activity counts between the groups (data not shown).

**Table 1:** Effects of KE concentration on energy expenditure.

**EE (kcal/day)**	**CON**	**KE5**	**KE10**	**KE15**	**KE20**	**KE25**	**KE30**
REE	8.1 ± 0.3	8.0 ± 0.3	7.2 ± 0.3	7.4 ± 0.3	7.1 ± 0.3	6.5 ± 0.3^†^	5.2 ± 0.8^†^
adjusted LSM	7.6 ± 0.3	7.3 ± 0.3	6.9 ± 0.2	7.3 ± 0.2	7.4 ± 0.3	6.9 ± 0.2	6.2 ± 0.3^*^
TEE	10.9 ± 0.3	10.9 ± 0.3	10.3 ± 0.3	10.3 ± 0.3	9.9 ± 0.3	9.5 ± 0.3^†^	8.1 ± 0.3^†^
adjusted LSM	10.4 ± 0.2	10.2 ± 0.2	9.7 ± 0.2^*^	10.1 ± 0.2	10.2 ± 0.2	10.0 ± 0.2	9.4 ± 0.2^†^
Light EE	5.1 ± 0.1	5.1 ± 0.1	4.7 ± 0.1	4.5 ± 0.1	4.5 ± 0.2	4.3 ± 0.1^†^	3.8 ± 0.1^†^
adjusted LSM	4.9 ± 0.1	4.8 ± 0.1	4.5 ± 0.1	4.4 ± 0.1^‡^	4.6 ± 0.1	4.5 ± 0.1	4.3 ± 0.1
Dark EE	5.8 ± 0.2	5.8 ± 0.2	5.5 ± 0.2	5.7 ± 0.2	5.4 ± 0.2	5.1 ± 0.2^*^	4.2 ± 0.2^†^
adjusted LSM	5.5 ± 0.1	5.3 ± 0.1	5.2 ± 0.1	5.6 ± 0.1	5.6 ± 0.1	5.4 ± 0.1	5.0 ± 0.1^‡^

*Data are presented as mean ± SE*.

* *Significantly different from CON (P < 0.05)*,

† *(P < 0.01)*,

‡ *(P = 0.051)*.

*EE, Energy Expenditure; LSM, least square means—adjusted for lean mass and fat mass*.

## Discussion

The ketogenic diet has been shown to decrease appetite (2) and attenuate reductions in EE that accompany weight loss in humans (3). In rodents, the ketogenic diet produces thermogenic responses in brown adipose tissue (9) that are consistent with increased EE despite weight loss (10, 11). These findings highlight the possibility that circulating concentrations of βHB and AcAc may contribute to the beneficial effects of a ketogenic diet on components of energy balance and weight loss. We are unaware of any reports that have examined concentration-dependent effects of exogenous ketones on components of energy balance.

This study aimed to determine the concentration-dependent effect of an isocaloric KE-supplemented standard chow diet on BW, body composition, and EE in lean, male C57BL/6J mice. Our results showed mice receiving the highest KE dose (KE30) had a reduction in food intake, BW, FM, and LBM, as well as decreased REE and TEE compared to CON. Surprisingly, the KE20 and KE25 mice consumed the same amount of food as CON, but had decreased BW (KE25 only), FM, and preserved LBM. Furthermore, after adjustment for LBM and FM, there was no difference in REE and TEE in these two groups compared to CON. These findings suggest that a diet consisting of 20–25% BD-AcAc_2_ may be an optimal formulation for future studies when examining the effects of KE on components of energy expenditure or loss.

The control of food intake is influenced by a balance between hunger and satiety and closely regulated by the central nervous system. Evidence from ketogenic diet studies in humans report decreased hunger (12, 13) that may be attributed to a suppression of ghrelin (14), but the mechanisms behind this response are not yet completely understood. Under conditions of starvation or with a ketogenic diet, βHB and AcAc are elevated by metabolism of fatty acids in the liver from adipose tissue and transported to the brain (and other extrahepatic tissues) to be used for energy (15, 16). Thus, it is possible that the consumption of exogenous ketones could provide similar benefits as endogenous ketones on appetite and body weight regulation without increasing circulating fatty acids seen with a ketogenic diet. In this study, food intake decreased only with KE30, corroborating our previous findings of 30% BD-AcAc_2_ on food intake (8). Thus, the effect of exogenous ketones on attenuating food intake appears to be threshold-dependent at a concentration of 30% or higher. Further research is needed to pursue these questions.

Dietary KE have consistently been shown to induce weight loss (4, 7, 8, 17), or prevent weight gain (5), but only two studies have reported changes in EE (8, 17). Increased REE was reported with D-β-hydroxybutyrate-1,3-butanediol (17), and we reported both increased REE and TEE in mice fed a high-fat diet+BD-AcAc_2_ compared to pair-fed high-fat diet controls (8). In the current investigation, we found no differences in EE among any of the KE groups compared to CON, except for KE30 which had decreased REE and TEE and KE10 which had decreased TEE, but not REE. Given that KE30 mice weighed the least, had decreased FM and LBM, and consumed the least amount of energy, our results of lowered REE and TEE coincides with studies of caloric restriction and energy conservation (18). The most likely explanation for lower TEE in the KE10 group compared to CON would be lower physical activity. Exploration of horizontal and vertical physical activity revealed no differences but a small, but statistically insignificant lower energy intake, may have contributed. However, the KE20 and KE25 mice had similar food intake and EE as CON, but significantly lower BW and FM, suggesting that a portion of the energy consumed in the KE diet was inefficiently absorbed and metabolized, or that the diet resulted in adipose tissue catabolism through currently unknown mechanisms. Future investigations are required to examine the metabolic fate and subsequent effects of acetoacetate and βHB on energy balance. A limitation of this study was that we did not measure circulating βHB or AcAc concentrations and this will be required in future studies for safety and tolerability.

## Conclusions

Translation of preclinical findings in rodents to application in humans requires the identification of concentration-dependent effects of exogenous ketones on components of energy balance and adiposity. Dietary consumption of the KE BD-AcAc_2_ decreased food intake at a threshold of 30%, but produced significant changes in BW and adiposity starting at 20%. Neither energy intake nor energy expenditure were different among the KE20 or KE25 groups, suggesting that dietary energy was inefficiently absorbed or metabolized.

## Ethics Statement

The study was approved by the UAB Institutional Animal Care and Use Committee (IACUC) and all conditions conformed to the care procedures employed by the UAB Animal Resources Program.

## Author Contributions

SED, RD, AK, AP, DA, DD, and EP designed the study. SED, RD, and EP performed data collection. SED, RD, SLD, DA, and EP contributed to data analysis. All authors contributed to writing and reviewing the manuscript. All authors have approved the final version of this article.

### Conflict of Interest Statement

DD, AP, AK are inventors on patents related to exogenous ketones, including BD-AcAc_2_. AP is a scientific advisor to Pruvit, and owner of Poff Medical Consulting and Communications, LLC and Metabolic Health Initiative, LLC. DD is an owner of Ketone Technologies, LLC. The remaining authors declare that the research was conducted in the absence of any commercial or financial relationships that could be construed as a potential conflict of interest.
