# Supplementary material for: Concentration-Dependent Effects of a Dietary Ketone Ester on Components of Energy Balance in Mice
Source: Front Nutr. 2019 May 1;6:56. doi: 10.3389/fnut.2019.00056 (PMC6504762; doi:10.3389/fnut.2019.00056)
Supplement: Supplementary file 1 [file Table_1.pdf]

Supplemental Table 1.

Supplemental Table 1. AIN93-G Purified Diet with Added Ketone Ester by % Calorie

Ingredient	Diet (kcal/g)						
	**CON**	**KE5**	**KE10**	**KE15**	**KE20**	**KE25**	**KE30**
Casein	0.716	0.716	0.716	0.716	0.716	0.716	0.716
L-cysteine	0.012	0.012	0.012	0.012	0.012	0.012	0.012
Sucrose	0.400	0.400	0.400	0.400	0.400	0.400	0.400
Cornstarch	1.431	1.244	1.056	0.868	0.680	0.492	0.308
Dyetrose	0.502	0.502	0.502	0.502	0.502	0.502	0.502
Soybean Oil	0.630	0.630	0.630	0.630	0.630	0.630	0.630
t-butylhydroquinone	0	0	0	0	0	0	0
Cellulose	0	0	0	0	0	0	0
Mineral Mix #210025	0.031	0.031	0.031	0.031	0.031	0.031	0.031
Vitamin Mix #310025	0.039	0.039	0.039	0.039	0.039	0.039	0.039
Choline Bitartrate	0	0	0	0	0	0	0
Sodium Saccharin	0	0	0	0	0	0	0
Ketone Ester	0	0.188	0.376	0.564	0.752	0.940	1.128
**TOTAL (kcal/g food)**	**3.761**	**3.762**	**3.762**	**3.762**	**3.762**	**3.762**	**3.766**
% Carbohydrate	62.9	57.7	52.5	47.3	42.1	36.9	31.7
% Fat	16.7	16.7	16.7	16.7	16.7	16.7	16.7
% Protein	20.3	20.3	20.3	20.3	20.3	20.3	20.3
% Ketone Ester	0	5.0	10.0	15.0	20.0	25.0	30.0
